# Association of Single Nucleotide Polymorphisms in TCF2 with Type 2 Diabetes Susceptibility in a Han Chinese Population

**DOI:** 10.1371/journal.pone.0052938

**Published:** 2012-12-31

**Authors:** Xuelong Zhang, Hong Qiao, Yanling Zhao, Xi Wang, Haiming Sun, An Liu, Lidan Xu, Donglin Sun, Yan Jin, Yang Yu, Xiangning Meng, Jing Bai, Feng Chen, Songbin Fu

**Affiliations:** 1 Laboratory of Medical Genetics, Harbin Medical University, Harbin, China; 2 Department of Endocrinology, the Second Affiliated Hospital, Harbin Medical University, Harbin, China; 3 Department of Gastroenterology, the First Affiliated Hospital, Harbin Medical University, Harbin, China; 4 Key Laboratory of Medical Genetics, Harbin Medical University, Heilongjiang Higher Education Institutions, Harbin, China; Kunming Institute of Zoology, Chinese Academy of Sciences, China

## Abstract

Hepatocyte nuclear factor 1β (HNF1β), a transcription factor encoded by the transcription factor 2 gene (TCF2), plays a critical role in pancreatic cell formation and glucose homeostasis. It has been suggested that single nucleotide polymorphisms (SNPs) of TCF2 are associated with susceptibility to type 2 diabetes (T2D). However, published results are inconsistent and inclusive. To further investigate the role of these common variants, we examined the association of TCF2 polymorphisms with the risk of T2D in a Han population in northeastern China. We genotyped five SNPs in 624 T2D patients and 630 healthy controls by using a SNaPshot method, and evaluated the T2D risk conferred by individual SNPs and haplotypes. In the single-locus analysis, we found that rs752010, rs4430796 and rs7501939 showed allelic differences between T2D patients and healthy controls, with an OR of 1.26 (95% CI 1.08–1.51, *P = *0.003), an OR of 1.23 (95% CI 1.06–1.55, *P = *0.001) and an OR of 1.28 (95% CI 1.10–1.61, *P = *0.001), respectively. Genotype association analysis of each locus also revealed that the homozygous carriers of the at-risk allele had a significant increased T2D risk compared to homozygous carriers of the other allele (OR 1.78, 95% CI 1.20–2.64 for rs752010; OR 1.82, 95% CI 1.24–2.67 for rs4430796; OR 1.95, 95% CI 1.31–2.90 for rs7501939), even after Bonferroni correction for multiple comparisons. Besides, the haplotype-based analysis demonstrated that AGT in block rs752010-rs4430796-rs7501939 was associated with about 30% increase in T2D risk (OR 1.31, 95% CI 1.09–1.57, *P = *0.01). Our findings suggested that TCF2 variants may be involved in T2D risk in a Han population of northeastern China. Larger studies with ethnically diverse populations are warranted to confirm the results reported in this investigation.

## Introduction

Although the exact etiology is unknown, type 2 diabetes (T2D) is characterized by dysfunction of pancreatic beta cells and insulin resistance, resulting from the complex interactions of multiple genetic variants and environmental factors including diet, exercise, stress, and medical treatment [1], [2]. In recent years, important advances for the genetic determinants of T2D have come with the implement of genome-wide association studies (GWAS). To date, at least 44 T2D susceptibly variants have been identified. The marked increase in loci associated with T2D is owed to the advent of GWAS [3]. An interesting observation is that specific variants in the TCF2 gene have been identified to be associated with both the risk of prostate cancer [4], [5], [6] and the risk of T2D [4], [7] with the effects being in the opposite direction for these two phenotypes.

Human transcription factor 2 gene (TCF2) consists nine exons and encodes the 557-amino hepatocyte nuclear factor 1β (HNF1β). HNF1β is a POU (Pit-1Oct-1/2-UNC-86) domain transcription factor closely related to HNF1α. Both are expressed in the pancreas, liver, and kidney [8], [9]. Mutations in TCF2 were initially described in a monogenic form of diabetes, namely maturity onset diabetes of the young type 5 (MODY5) [10]. In humans, TCF2 variants are also associated with several diseases such as defective kidney development [11], disturbed liver function [12], pancreas atrophy [13], defective insulin secretion [14], malformations of the genital tract [15], [16] and diverse cancers [17]. The broad spectrum of HNF1β affected phenotypes is possibly also linked to its ability to interact with many other regulatory molecules [18].

In recent years, there are several studies explored the relationship of TCF2 polymorphisms and T2D. Some studies have indicated that SNPs on TCF2 were associated with the risk of T2D in Swedish, Finnish and Canadian populations [7], [19]. The association signals were located on intron 2 (rs757210), intron 6 (rs1008284) and intron 8 (rs3110641). Moreover, a genome-wide association study identified that the G allele of rs7501939 (in intron 1) and A allele of rs4430796 (in intron 2) were associated with reduced risk of T2D in Caucasians and Africans [4]. Similarly, one Chinese study using a southern population observed the risk G allele of rs4430796 was significantly associated with T2D [20]. However, another Caucasian analysis using the subjects from Sweden and Finland could not replicate the association of TCF2 loci with future risk of T2D in two prospective studies [21]. The major reasons for this discrepancy may be related to ethnicity and/or environmental factors. Population substructure was also a potentially important cause of different results in case-control genetic studies [22]. To improve our understanding of the role of TCF2 in T2D predisposition, it is extremely important to understand the consequences of inheriting the variants in other ethnic populations. Therefore, in the present study, we conducted a case-control study to investigate the role of TCF2 SNPs on T2D risk in a Han population in northeastern China.

## Materials and Methods

### Ethics Statement

A standard informed written consent procedure was included in the protocol, and was reviewed and approved by the Ethics Committee of Harbin Medical University, China. Participants gave their written consent after the nature of study had been fully explained.

### Participants

A total of 1,254 Han Chinese were enrolled in the current study from Harbin, northeast of China. The study individuals must be stable residents in the areas. “Stable residents” refer that all subjects are of northern Chinese Han ancestry and reside in Harbin area for at least three generations. The participants should not be genetically related for at least three generations.

The DNA and serum samples of 624 unrelated type 2 diabetic individuals (419 men, 205 women; age 51.58±12.66 years) and 630 control individuals (423 men, 207 women; age 51.38±12.45 years) were used in the present investigation. The T2D patients were recruited from the inpatient department of affiliated second hospital, Harbin Medical University. T2D was confirmed by scrutinizing medical records for symptoms, use of medication and measuring fasting glucose levels following the WHO criteria [23]. Exclusion criteria for cases were individuals with diabetes caused by: liver dysfunction; malignancy; steroids and other drugs that might raise glucose levels; and monogenic disorder known to cause diabetes. Type 1 diabetes and mitochondrial diabetes were also excluded. Moreover, more than 50% of T2D patients were diagnosed at the age of 45–55 years and have 5–10 years duration of disease. Controls with a fasting plasma glucose (FPG) concentration <6.1 mmol/l and 2-h plasma glucose <7.8 mmol/l were enrolled from an annual health check conducted at the same hospital, who had no family history of T2D. Body mass index (BMI) was calculated as (weight (kg)/height (meter) ^2^). Data are showed as means±SD (Table 1).

**Table 1:** Clinical characteristics of the study participants.

Characteristic	Type 2 diabetes	Controls
Number	624	630
Men/women (n/n)	419/205	423/207
Age (years)	51.58±12.66	51.38±12.45
BMI (kg/m^2^)	25.32±4.23	24.18±3.69
Fasting blood glucose (mmol/l)	10.06±3.53	4.80±0.49

Data are means±SD.

### Genotyping

Genomic DNA was extracted from peripheral blood leukocytes using the QIAamp DNA Blood Kit (Valencia, CA, USA). In the present study, we genotyped five SNPs (rs3110641, rs1008284, rs752010, rs4430796 and rs7501939) which previously reported to be associated with T2D in various populations using SNaPshot assay. All the SNPs were in intronic region and there was no established nonsynonymous SNP in TCF2. Primers to amplify a different-sized fragment for each SNP within a multiplex were designed using Primer3 (http://frodo.wi.mit.edu/) and extension primers again differing in length within a multiplex were picked from the sequence immediately up- or down-stream of the SNP. Primer interactions within the multiplex were evaluated and minimized using the AutoDimer program (http://www.cstl.nist.gov/div831/strbase/AutoDimerHomepage/AutoDimerProgramHomepage.htm). PCRs consisting of 10–50 ng DNA, 1 X HotStarTaq buffer, 3 mM MgCl_2_, 300 µM of each dNTP, 0.08 µM of each primer and one unit of HotStarTaq polymerase (Qiagen) were set up in a 20 µl reaction volume. A touchdown PCR program was used: denaturation at 95°C for 15 min, then 11 cycles of 94°C for 20 sec, annealing at 65°C for 40 sec and extension at 72°C for 90 sec, decreasing the annealing temperature by 0.5°C per cycle. This was followed by 24 cycles of denaturation at 94°C for 20 sec, annealing at 59°C for 30 sec and extension at 72°C for 90 sec and a final extension at 72°C for 5 min. The PCR products were purified by treatment with Exonuclease I (USB Corporation) and Shrimp Alkaline Phosphatase (USB Corporation) at 37°C for 1 hr followed by 80°C for 15 min. The extension reaction contained 1 X ABI Prism SNaPshot Multiplex ready reaction mix (Applied Biosystems), 0.5 µM of each primer and 1 µl of each PCR product and was carried out as recommended (Applied Biosystems). The extended PCR products were purified using 1 unit Shrimp Alkaline Phosphatase, then run on an ABI 3130XL Genetic Analyzer. SNP calling was carried out using GeneMapper™ software v.4.0 (Applied Biosystems). For quality control, genotyping was done without knowledge of case/control status of the subjects, and a 5% random sample of cases and controls was genotyped twice per all SNPs by different persons. In detail, we included 70 pairs of blind duplicates, and observed zero discrepancy in 350 comparisons. The reproducibility was 100%.

### Statistical Analysis

The Hardy-Weinberg equilibrium was evaluated using Pearson’s χ^2^ test separately for cases and controls. Differences between cases and controls in demographic characteristics and risk factors were evaluated by χ^2^ test (for categorical variables) or Student’s *t*-test (for continuous variables). The allele frequencies between cases and controls were compared using a χ^2^ test or Fisher’s exact probability test, where appropriate. Statistical evaluations for testing genetic effects of association between the case-control status and each individual SNP, measured by the odds ratios (ORs) and 95% confidence intervals (CIs), were estimated using unconditional logistic regression after adjusting for age, gender and BMI. Genotypes distributions between cases and controls were determined by logistic regression under additive, dominant and recessive model, respectively. Statistical analyses were performed using SPSS for Windows software (version 17.0; SPSS, Chicago, IL). We analyzed the data using two-sided *P* values. We also applied Bonferroni’s correction for multiple testing.

We examined the degree of LD among markers to partition haplotype blocks using the solid spine of LD algorithms in Haploview V4.1. This internally developed method searches for a “spine” of strong LD running from one marker to another along the legs of the triangle in the LD chart. In the within-block region of TCF2, we applied the haplotype-trait association test by Haplo.Stats (http://mayoresearch.mayo.edu/mayo/research/schaid_lab/software.cfm) [24]. Common haplotypes with frequencies of greater than 0.01 were compared between cases and controls. Adjusted ORs and 95% CIs were also generated for each haplotype compared to the most common haplotype, based on the generalized linear model framework in the method, which allows adjustment for confounding variables.

Statistical power was assessed using the Genetic Power Calculator [25]. Considering 0.6% prevalence of the disease, a risk allele frequency of 25%, and an additive genetic model, we had at least 80% power to detect an OR of 1.25 at the 0.05 level.

## Results

### Subject Characteristics

The subjects including 624 T2D patients and 630 non-diabetic controls were genotyped for five representative TCF2 SNPs and analyzed for association with T2D. The case-control cohort used in this investigation was matched for ethnicity, culture and geographical locations. The baseline clinical characteristics of the subjects are shown in Table 1. A slightly higher proportion of males than females were observed in both groups, which may be due to a participation bias. The BMI between the two groups were significantly different (25.32±4.23 vs. 24.18±3.69 km/m^2^, *P*<0.001). The FPG level was also significantly higher in the diabetic group subjects compared to normal control subjects (10.06±3.53 vs. 4.80±0.49, *P*<0.001).

### Analysis of Associations with Individual SNPs

The information on the genomic location of the investigated SNPs and minor allele frequencies is summarized in Table 2. Genotype distributions of the all the investigated SNPs did not deviated from Hardy-Weinberg equilibrium in both cases and controls. As shown in Table 2, rs752010, rs4430796 and rs7501939 showed allelic difference between case and control groups in the northern Chinese Han population, with an OR of 1.29 (95% CI 1.09–1.53, *P = *0.003), an OR of 1.31 (95% CI 1.11–1.55, *P = *0.002) and an OR of 1.32 (95% CI 1.12–1.57, *P = *0.001), respectively. In addition, the risk alleles of these three SNPs were A, G and T in our population, consistent with previous reports in European, African and Asian populations [4], [7], [19], [20]. After adjusted for sex, age and log-transformed BMI by logistic regression analysis, the three SNPs conferred independent risks for the disease (OR 1.26, 95% CI 1.08–1.51, *P = *0.003; OR 1.23, 95% CI 1.06–1.55, *P = *0.001; and OR 1.28, 95% CI 1.10–1.61, *P = *0.001). Furthermore, the association still stood after Bonferroni’s correction (corrected *P<*0.01). However, we failed to replicate the effect for the other two SNPs (rs3110641 and rs1008284) which were identified in the Caucasian populations (*P*>0.05).

**Table 2:** Association analysis of TCF2 candidate SNPs for type 2 diabetes.

SNP	chromosomeposition	TCF2 region	Major/Minorallele	Risk allele	Risk allele frequency		Allele-specific		Allele-specific (adjusted)		*P* for HWET
					case	control	*P* value	OR (95% CI)	*P* value*	OR (95% CI)	
rs3110641 [7]	36047417	Intron 8	C/T	C	0.752	0.743	0.58	1.05 (0.88–1.26)	0.56	1.08 (0.90–2.31)	0.27
rs1008284 [19]	36062458	Intron 6	C/T	C	0.757	0.740	0.31	1.10 (0.92–1.31)	0.30	1.14 (0.89–1.35)	0.63
rs752010 [7]	36096515	Intron 2	G/A	A	0.338	0.283	0.003	1.29 (1.09–1.53)	0.003	1.26 (1.08–1.51)	1.00
rs4430796 [4]	36098040	Intron 2	A/G	G	0.353	0.294	0.002	1.31 (1.11–1.55)	0.001	1.23 (1.06–1.55)	0.98
rs7501939 [4]	36101156	Intron 1	C/T	T	0.328	0.269	0.001	1.32 (1.12–1.57)	0.001	1.28 (1.10–1.61)	0.22

* Adjusted for age, sex and log_e_ BMI.

We further analyzed the effect of the genotypes of five SNPs under three different genetic models by logistic tests (Table 3). The rs752010, rs4430796 and rs7501939 were observed to be associated with T2D risk by both dominant and recessive model analyses (OR 1.31–1.80, *P<*0.05), though these associations did not remain significant after correction for multiple testing. Meanwhile, our analysis revealed that three positive associations under additive model were more evident than those under dominant and recessive models. For each positive association SNP, the homozygous genotype of risk allele conferred a significantly increased risk for T2D compared to the homozygous genotype of the common wild-type allele (adjusted OR 1.78, 95% CI 1.20–2.64 for rs752010; adjusted OR 1.82, 95% CI 1.24–2.67 for rs4430796; adjusted OR 1.95, 95% CI 1.31–2.90 for rs7501939). The associations still stood after Bonferroni’s correction (corrected *P<*0.004). However, no association was observed in any genetic models for the rest two SNPs (*P*>0.05).

**Table 3:** Effects of TCF2 genotypes on the risk of type 2 diabetes under different genetic models.

SNP	Genotype	Additive model		Dominant model		Recessive model	
		*P* value	OR (95% CI)	*P* value	OR (95% CI)	*P* value	OR (95% CI)
rs3110641	TT			0.13	0.69 (0.43–1.12)	0.17	1.17 (0.94–1.46)
	CT	0.05	0.61 (0.37–1.00)				
	CC	0.27	0.76 (0.46–1.24)				
rs1008284	TT			0.70	0.92 (0.59–1.43)	0.15	1.18 (0.94–1.47)
	CT	0.39	0.81 (0.51–1.30)				
	CC	0.97	0.99 (0.63–1.56)				
rs752010	GG			0.01	1.32 (1.06–1.65)	0.01	1.61 (1.10–2.35)
	AG	0.08	1.23 (0.98–1.56)				
	AA	0.003	1.78 (1.20–2.64)				
rs4430796	AA			0.01	1.34 (1.07–1.67)	0.008	1.63 (1.13–2.36)
	AG	0.07	1.24 (0.98–1.57)				
	GG	0.002	1.82 (1.24–2.67)				
rs7501939	CC			0.02	1.31 (1.05–1.63)	0.004	1.80 (1.23–2.64)
	CT	0.14	1.19 (0.94–1.51)				
	TT	0.001	1.95 (1.31–2.90)				

*P* values and OR values were adjusted for age, sex and log_e_ BMI.

### Analysis of Haplotype Associations

**Figure 1:**
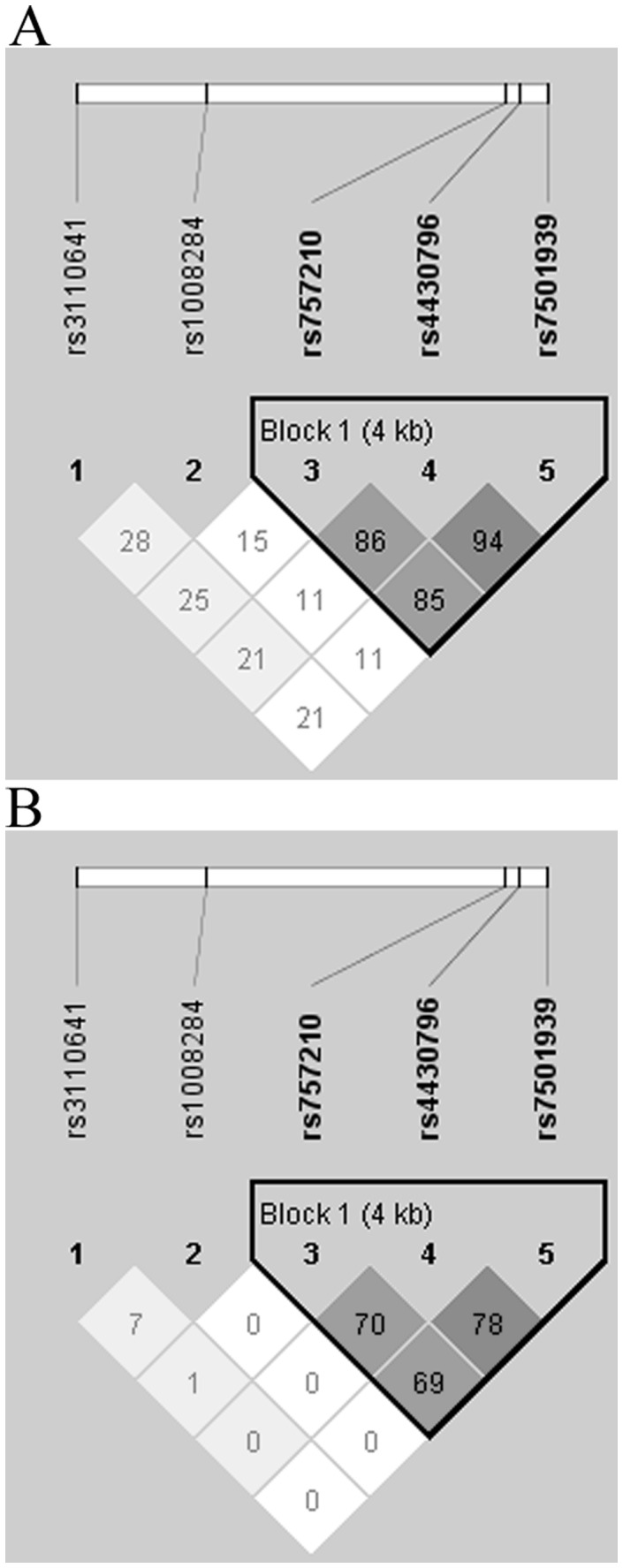
Linkage disequilibrium patterns of five typed SNPs in the Chinese population. This plot was generated using the Haploview program with the confidence intervals setting. One block was determined. The rs number (top: from left to right) corresponds to the SNP name and the number in each square is D′ values (A) or r^2^ values (B) between a pair of SNPs. The measure of LD (D′ or r^2^) among all possible pairs of SNPs is shown graphically according to the shade of color, whereas white represents very low LD values and dark represents very high LD values.

Haplotypes were constructed on the basis of the genotype data from five common SNPs using Haploview software, and pairwise linkage disequilibrium D′ values between SNPs and the reconstructed LD plots of the five SNPs in our subjects are shown in Figure 1. Three SNPs (in the order of rs752010, rs4430796 and rs7501939) were in strong LD with each other and therefore formed a haplotype block. Seven haplotypes that had a frequency of >1% were found (Table 4). Haplotype-specific analysis revealed that the haplotypes containing three risk alleles “AGT” were significantly associated with an enhanced risk of T2D compared with the most common haplotype “GAC” (adjusted OR 1.31, 95% CI 1.09–1.57, *P = *0.01). Moreover, the “AGT” haplotype remained significantly different between cases and controls after correction with a permutation test with 10,000 repetitions for multiple testing. There were no differences in the frequencies of other haplotypes between cases and controls and no significant association with T2D risk (*P*>0.05).

**Table 4:** Association of TCF2 rs752010-rs4430796-rs7501939 haplotypes with type 2 diabetes.

Haplotypes	Frequency	Haplotype frequenciesin the cases	Haplotype frequenciesin the controls	*P* value of χ^2^-test	OR (95% CI)
GAC	0.637	0.602	0.672	0.001	1.00 (Reference)
AGT	0.268	0.290	0.247	0.01	1.31 (1.09–1.57)
AAC	0.028	0.030	0.026	0.55	1.29 (0.79–2.08)
GGC	0.023	0.024	0.023	0.84	1.18 (0.70–1.98)
GGT	0.018	0.023	0.014	0.10	1.83 (1.01–3.31)
AGC	0.014	0.016	0.011	0.23	1.68 (0.85–3.34)
GAT	0.010	0.013	0.008	0.29	1.70 (0.78–3.71)

### Associations between SNPs and T2D Characteristics

In subjects with normal glucose regulation, the five SNPs were not associated with plasma glucose levels; insulin sensitivity or insulin secretion; adiposity-related anthropometrics such as BMI, waist circumference, and waist-to-hip ratio; and lipid-related traits (data not shown).

## Discussion

In this hospital-based case-control study in a Chinese Han population, we evaluated the association between the five SNPs of the TCF2 gene and T2D susceptibility. Three of the variants (rs752010, rs4430796 and rs7501939) had a significant association with T2D risk. The positive association of these variants remained significant after applying the Bonferroni correction (*P = *0.003, *P = *0.001 and *P = *0.001, respectively). In addition, haplotype analyses consistently revealed a statistically significant association between the haplotype “AGT” containing three risk alleles and enhanced risk of T2D compared with the most common haplotype “GAC”. When adjustments for multiple comparisons were made, the significant association was still noted. These findings support our hypothesis that genetic variants of the TCF2 gene may contribute to T2D susceptibility.

A number of studies have investigated a role of TCF2 common variations in Caucasian T2D with conflicting results. Bonnycastle et al. observed that rs1008284 (in intron 6) was associated with T2D in Finns [19]. Winckler et al. reported that rs757210 (in intron 2) and rs3110641 (in intron 8) were associated with T2D in a large Caucasian sample [7]. Gudmundsson et al. showed that rs7501939 (in intron 1) and rs4430796 (in intron 2) were associated with the disease in the Icelandic population [4]. However, another Caucasian analysis using populations of Sweden and Finland could not replicate the association of TCF2 loci with future risk of T2D in two prospective studies [21]. The possible reason for this inconsistence within Euro-Caucasians could be due to the genetic heterogeneity and different linkage disequilibrium structures existing among “Caucasians” because of quite different geographic locations and genetic origin. An association does not necessarily mean the nucleotide change at the associated SNP leads to direct effects on the phenotype under study; it’s likely that in many cases the associated SNP acts as markers linked to the causal genomic variants [26]. Therefore, it is likely that some causal variants could be ethnic-specific or could be present elsewhere in the same or nearby gene. It has been suggested that differences in the patterns of linkage disequilibrium between these SNPs and functional variants at these loci could underlie these disparate findings. Alternatively, gene-environment interactions may operate in the pathogenesis of T2D and that differences in the level of environmental risk factors in different populations may alter the impact of susceptibility loci on the risk of T2D.

Previous studies based on analysis of anatomical, archeological, linguistic and genetic data have consistently suggested the presence of a significant boundary between northern and southern populations in China, with the Yangtze River as the geographical boundary [27]. The Han Chinese population, although seemingly homogeneous, exhibits a complicated substructure as the genetics of northern Han Chinese differ greatly from those of southern Han Chinese [27]. Accordingly, a previous study suggested that the significant differences among northern and southern Han Chinese subpopulations should be carefully examined, especially when sample sources are diverse, as they may influence association studies [28]. One Chinese study using tag SNPs approach observed the risk G allele of rs4430796 was significantly associated with T2D in a southern population [20], which was consistent with our results. They also found that rs752010 as an associated SNP in the same direction with ours, although the association was not significant after a correction for multiple testing. In the current sample of northern Han Chinese, false-positive or false-negative associations owing to population substructure are less likely to exist. Our carefully ascertained, relatively homogeneous case-control sample of northern Han Chinese belongs to a single geographic location of Harbin city and must be stable residents in the area. Taken together, although the three variants were not necessarily the functional culprits, the solidly positive findings of these two association studies provided support for the hypothesis that the TCF2 gene was an important contributor to T2D in Chinese.

Our study has several strengths, including the study individuals from homogeneous populations of the same ethnicity and the consistency in allelic, genotypic and haplotypic associations of rs752010, rs4430796 and rs7501939 with T2D risk. To our knowledge, this study is the first to demonstrate that the TCF2 polymorphisms are associated with T2D in the northeastern Han Chinese. However, several inherent limitations and must be noted. First, because controls were collected from hospitals, some level of selection bias could not be ruled out. However, all control subjects in our study were those who came to hospitals for routine health examination but not hospitalized patients with specific diseases, probably making the controls more representative of the general population. In this condition, the potential selection bias was believed to be minimized. Second, we didn’t treat age at diagnosis as a dichotomous trait to stratify the T2D patients as a previous study [20], because the sample size might severely limit statistical power for subgroup analysis. Further well-designed investigations with larger sample sizes are warranted to confirm our findings.

### Conclusions

In conclusion, our comprehensive analysis of SNPs in the TCF2 gene suggested that TCF2 genotypes and haplotypes were associated with T2D risk. Further large scale association studies and functional studies will be useful to replicate the promising findings and to fully delineate the role of TCF2 in the pathogenesis of T2D.
